# UBE2C promotes the proliferation of acute myeloid leukemia cells through PI3K/AKT activation

**DOI:** 10.1186/s12885-024-12212-x

**Published:** 2024-04-18

**Authors:** Li Wang, Shuqin Zhao, Yongling Wang, Jianying Liu, Xiaoli Wang

**Affiliations:** 1 Department of Pediatrics, Yantai Yuhuangding Hospital, No. 20 Yudong Road, Zhifu District, Yantai City, Shandong 264099 China; 2 Department of Pediatrics, Yantai Yuhuangding Hospital Laishan Branch, No. 59, Shuanghe West Road, Laishan District, Yantai City, Shandong 264099 China

**Keywords:** Leukemia, UBE2C, PI3K/AKT pathway, Ferroptosis, Cell proliferation

## Abstract

This study aims to investigate the role and mechanism of tubiquitin-conjugating enzyme E2 C (UBE2C) in acute myeloid leukemia (AML). Initially, UBE2C expression in leukemia was analyzed using the Cancer Genome Atlas database. Further, we silenced UBE2C expression using small-hairpin RNA (sh-RNA). UBE2C expression was detected via the quantitative reverse-transcriptase polymerase chain reaction (qRT-PCR) and Western blot analysis. Apoptotic events and reactive oxygen species (ROS) levels were detected by flow cytometry. A xenograft model of leukemia cells were established, and the protein levels of UBE2C, KI-67, and cleaved-caspase 3 were detected by immunohistochemistry. We reported an overexpression of UBE2C in leukemia patients and cell lines (HL60, THP-1, U937, and KG-1 cells). Moreover, a high expression level of UBE2C was correlated with a dismal prognosis in AML patients. UBE2C knockdown inhibited the viability and promoted apoptosis in AML cells by regulating the phosphatidylinositol-3-kinase (PI3K)/protein kinase B (AKT) signaling pathway. Furthermore, UBE2C knockdown increased cellular Fe^2+^ and ROS levels, and enhanced erastin-induced ferroptosis in a proteasome-dependent manner. UBE2C knockdown also suppressed the tumor formation of AML cells in the mouse model. In summary, our findings suggest that UBE2C overexpression promotes the proliferation and inhibits ferroptosis in AML cells by activating the PI3K/AKT pathway.

**Supplementary Information:**

The online version contains supplementary material available at 10.1186/s12885-024-12212-x.

## Introduction

Acute myeloid leukemia (AML) has emerged as one of the dreadful malignant neoplasms of myeloid stem cell precursors (erythrocytes, platelets, and leukocytes, in addition to B and T cells) [1]. Notably, it has been recognized that the incidence of AML shows a proportional increment with age. The etiology and pathogenesis of AML are often caused by various genetic mutations in the pluripotent malignant hematopoietic stem cells [2–4]. Based on the genetic mutation profile and the deregulation of certain signaling pathways, such as FLT3 internal tandem duplication (FLT3-ITD), c-KIT, PI3K/AKT pathway, and non-coding RNAs, different targeted therapies have been developed to induce tumor-specific killing effect in AML [5–7]. Despite the current advances in AML treatment using various therapeutic modalities, the long-term survival of AML patients remains poor due to the rapid development of drug resistance and the high recurrence rate after treatment [7, 8]. Therefore, the formulation of novel therapeutic strategies based on different modality of cell death induction is an attractive direction to overcome drug resistance.

The iron element plays a crucial role in several biological processes such as mitochondrial respiration, immune surveillance, cell proliferation, and various metabolic activities. However, excessive accumulation of iron in different organs often leads to iron-dependent programmed cell death, known as ferroptosis. This process is characterized by the buildup of intracellular reactive oxygen species (ROS) and the end products of lipid peroxidation reactions [9]. Ample evidence has shown that ferroptosis can be employed in cancer treatment, since tumor cells tend to accumulate a higher level of intracellular iron for supporting the hyper-proliferation [10]. A previous study demonstrated that treating AML cells with erastin, a ferroptosis-based agent, significantly increased their sensitivity to different chemotherapeutic agents. These findings provide evidence supporting the potential of ferroptosis as a novel strategy for treating AML [11]. A growing body of evidence has highlighted the role of protein ubiquitination system in dictating ferroptosis sensitivity by targeting different ferroptosis regulators [12, 13]. These E3 ubiquitin ligases and deubiquitinating enzymes (DUBs) may serve as targets for modulating ferroptosis susceptibility.

The gene UBE2C encodes a protein called ubiquitin-conjugating enzyme E2 C, which has been found to regulate cell proliferation and cancer cell invasion [14]. This protein is involved in regulating the stability of cell cycle-related proteins, ultimately affecting cancer progression. Numerous studies have highlighted the significant role of UBE2C in tumor progression, such as in endometrial cancer [15], head and neck squamous cell carcinoma [16], breast cancer [17], and lung cancer [18]. Recently, an increasing number of studies demonstrated that UBE2C was highly expressed in various human malignancies, in which the high expression of UBE2C could be closely associated with tumor stage and clinical prognosis [17, 19, 20]. Nonetheless, the expression pattern of UBE2C in AML and its relationship with the prognosis of AML patients remained unexplored comprehensively towards correlating their expression with the cancer progression. It also remains elusive whether UBE2C impinges on ferroptosis susceptibility in AML cells.

Motivated by these considerations, this study aims to investigate the role of UBE2C in leukemia development and its underlying mechanism. To explore these aspects, we analyzed the expression levels of UBE2C in AML using the TCGA database, and confirmed the UBE2C expression pattern of UBEC2 in clinical samples. We further analyzed the correlation of UBE2C expression levels and the prognosis in AML patients. Additionally, we established a stable cell line with UBE2C knockdown using lentivirus. The effect of knocking down UBE2C on cell proliferation and survival was investigated in both the cell and animal models. We also examined the impact of UBE2C silencing on erastin-induced ferroptosis.

## Materials and methods

### Clinical samples

The study collected clinical samples from leukemia patients, along with paracancerous tissues (*n* = 50 pairs). Inclusion criteria were set as follows: patients with clinical symptoms of infection and anemia, and those aged over 18 years without prior radiotherapy or chemotherapy. Exclusion criteria were set as follows: patients combined with other blood disorders, patients with autoimmune diseases, and patients who were mentally incompetent to cooperate. The medical ethics committee of Yantai Yuhuangding Hospital approved the clinical study, and selected subjects were required to provide a signed informed consent form. The UBE2C expression in The Cancer Genome Atlas (TCGA)-acute leukemia was analyzed using the Gene Expression Profiling Interactive Analysis (GEPIA) database. Additionally, the Kaplan-Meier (KM) plotter was used to plot the survival curves of UBE2C expression and AML patients.

### Cell culture

The HL60, THP-1, U937, and KG-1 cell lines were obtained from the Shanghai Cell Bank of the Chinese Academy of Sciences (CAS, Shanghai, China). The cells were cultured in Roswell Park Memorial Institute (RPMI)-1640 Medium (Gibco, Waltham, USA) supplemented with 10% fetal bovine serum (FBS, Gibco) as a nourishing medium. The cell cultures were maintained in a 5% CO_2_ incubator at 37 °C.

### sh-UBE2C transfection

The lentiviral vector for UBE2C short hairpin ribose nucleic acid (shRNA) and the negative control (sh-NC) were constructed by Shanghai GeneKai Chemical Technology Co. Ltd. (Shanghai, China). Once the cell fusion reached 30-50%, the sh-RNA was diluted to a concentration of 50 nmol/L using Opti-MEM. The designed sh-RNA was then transfected into the seeded cells using Lipofectamine 2000 transfection complex (Thermo Fischer Scientific, Waltham, USA). After 4–6 h of transfection, the medium was replaced with fresh medium.

### qRT-PCR analysis

The total RNA was extracted from each group of cells, including the transfected cell lines, using the Trizol reagent according to the manufacturer’s instructions (Invitrogen, Waltham, USA). Subsequently, the complementary deoxyribonucleic acid (cDNA) was reverse transcribed using the TaqMan MicroRNA Reverse Transcription Kit (Life Technologies Co. Ltd., Carlsbad, USA). Quantitative real-time PCR was conducted with the SYBRGreen Mix Reagent (11201ES03, Shanghai Yisheng) in a Roche Light Cycler 480 fluorescence PCR instrument. The primer sequences are as stated below.

UBE2C - Forward: GACCTGAGGTATAAGCTCTCGC.

Reverse: CAGGGCAGACCACTTTTCCTT.

GAPDH - Forward: CTCCTCCTGTTCGACAGTCAGC.

Reverse: CCCAATACGACCAAATCCGTT.

Finally, the relative mRNA expression was analyzed by the 2^−ΔΔCT^ method using GAPDH as an internal reference.

### WB analysis

To determine the protein expression levels, the cells in each group were transferred to a 1.5 mL tube and centrifuged. The supernatant was then discarded and 200 µL of radioimmunoprecipitation assay (RIPA) lysate buffer (Solarbio Life Sciences, Beijing, China) was added for cell lysis on ice for 15 min. Afterward, 30 µL of the supernatant was centrifuged and the protein concentration was measured using a bicinchoninic acid (BCA) kit (Solarbio Life Sciences). Subsequently, 100 µL of protein supernatant was mixed with 25 µL of sodium dodecyl-sulfate (SDS) protein loading buffer (Solarbio Life Sciences) and heated in a metal water bath at 100 ℃ for electrophoresis. The gels were then transferred to a poly(vinylidene fluoride) (PVDF) membrane and incubated with 5% skimmed milk powder at room temperature for 1–2 h. Next, the blots were incubated with the primary antibodies of diluted GAPDH (1:1,000, ab8245), GPX4 (1:1,000, ab262509), SLC7A11 (1:1,000, ab275411), p-PI3K (1:1 000, ab140307), PI3K (1:1 000, ab302958), p-AKT (1:1 000, ab283852), and AKT (1:1 000, ab8805) (Abcam, Cambridge, UK) overnight at 4 ℃. After washing the membrane, the blot was incubated with the HRP-linked goat anti-rabbit immunoglobulin (IgG, SAB43714, Bioswamp, Wuhan, China) at room temperature for 2 h. The target proteins were detected by the electrochemiluminescence (ECL) method (Beyotime Biotechnology Co. Ltd., Shanghai, China).

### CCK-8 assay

The proliferation ability of cells in each group was assessed using the CCK-8 assay. Cells in the logarithmic phase were collected and seeded in 96-well plates at a density of 2 × 10^4^ cells per well in a volume of 200 µL. After the cells attached to the bottom of the plate, the CCK-8 reagent kit (Solarbio Life Sciences) was added at 0, 24, 48, and 72 h. The absorbance (OD) values of each well at 450 nm were measured following the manufacturer’s instructions.

### EdU staining

The proliferation rate of culture cells was qualitatively analyzed using the EdU staining method. Initially, each group of cells was seeded at a density of 2 × 10^4^ cells/well on a coverslip in a 6-well plate. They were then fixed with 500 µL of 40 g/L paraformaldehyde for 20 min at room temperature. Next, the cells were permeabilized by adding 500 µL of 3 mL/L Triton X-100 (solarbio, Beijing, China) for 20 min. After a wash with phosphate-buffered saline (PBS), each well received 86 µL of reaction buffer, 4 µL of CuSO4, 0.2 µL of azide, and 10 µL of buffer additive. The mixture was incubated for 30 min at room temperature in the darkness. The supernatant was then aspirated, and the nuclei were counter-stained with 4’,6-diamidino-2-phenylindole (DAPI) for 20 min. Following another wash with PBS, the stained cells were imaged using a fluorescent inverted microscope.

### Flow cytometry

Flow cytometric investigations were conducted to assess apoptosis. Cells at the logarithmic phase were initially seeded in 12-well plates at a density of 6 × 10^5^ cells per well. After transfection, the cells were collected by centrifugation, washed twice with PBS, and then Annexin V-fluorescein isothiocyanate (FITC) and propidium iodide (PI) staining solution (Pricalla, Wuhan, China) were added. The cells were incubated for 15 min at room temperature, protected from light. Subsequently, the stained cells were subjected to flow cytometric investigations to determine the apoptotic rates of the transfected cells. The apoptosis rate in the cell lines was determined based on the presence of annexin V+/PI- cells, which were considered as early apoptotic cells, and annexin V+/PI + cells, which were considered as late apoptotic cells.

### Fe^2+^ level detection

Intracellular Fe2 + levels in each group of cells were measured using the iron assay kit from Abcam Co. Ltd. (ab83366, UK). Standard and treatment wells were prepared separately. In the standard wells, 200 µL of standards with different concentrations were added to the corresponding wells of the enzyme labeling plate. Similarly, 200 µL of the sample was added to the corresponding wells of the enzyme plate. After that, 100 µL of reagent II was added to the mixture and incubated for 10 min at 37 °C. Finally, the OD value of each well was measured at 593 nm using an enzyme marker.

### Xenograft tumor model in nude mice

BALB/c nude mice (*n* = 10, male, 4–6 weeks, and 18–22 g) purchased from Beijing Vital River Laboratory Animal Technology Co., Ltd. (Beijing, China) were housed under specific pathogen-free (SPF) conditions. All animal-related experiments and protocols were approved by the animal ethics committee of Yantai Yuhuangding Hospital. Initially, HL-60 cells were collected and washed twice with PBS. The cells were then resuspended in serum-free medium and adjusted to a concentration of 1 × 10^7^ cells/100 µL. Next, the cells were mixed with an equal volume of Matrigel (BD356234, Corning, USA) and injected subcutaneously into the right axilla of nude mice. The subcutaneously grown tumor volumes were measured on days 4, 8, 12, 16, 20, 24, 28, 32, and 36 using the formula V=(L×W^2^)/2, where L represents the length of the tumor and W represents the width of the tumor. At the end of the experiment, the mice were euthanized using carbon dioxide asphyxiation (flow rate: 35–45% displacement of the chamber volume per min) for 10 min, followed by cervical dislocation. The death was confirmed by the lack of movement, the cessation of heartbeat and breath. The tumors were separated from the sacrificed animals and weighed. Finally, immunohistochemical staining was performed using the Image-Pro Plus 6.0 software.

### Immunohistochemical (IHC) staining

The collected tumor tissues from the sacrificed animals were fixed in a 10% v/v neutral formaldehyde solution (Sigma, St. Louis, USA) and then sectioned into thin sections. These tissue sections were dehydrated by treating them with xylene for 30 min. Subsequently, the tissue sections were incubated with primary antibodies of UBE2C, Ki-67, and cleaved-caspase 3 monoclonal antibodies (Dako, Copenhagen, Denmark) for 20 min. Following a wash with PBS, a horseradish peroxidase (HRP)-labeled goat anti-mouse secondary antibody was added and incubated at 37 ºC for 20 min. The DAB color development solution was then added after another wash with PBS, and the sections were sealed. Finally, the tissue sections were observed under a microscope to detect the expression of positive cells.

### Statistical analysis

The experimental data were expressed as mean ± standard deviation (x ± s). The data were analyzed using the SPSS19.0 statistical software, and independent samples *t*-test was used to compare the two groups. In addition, one-way analysis of variance (ANOVA) was used to compare multiple groups. KM-plotter was applied to plot the survival curves of patients with high- and low- expression AML groups of UBE2C. A difference of *P* < 0.05 was considered statistically significant.

## Results

### UBE2C is highly expressed in the leukemia cells

**Fig. 1:**
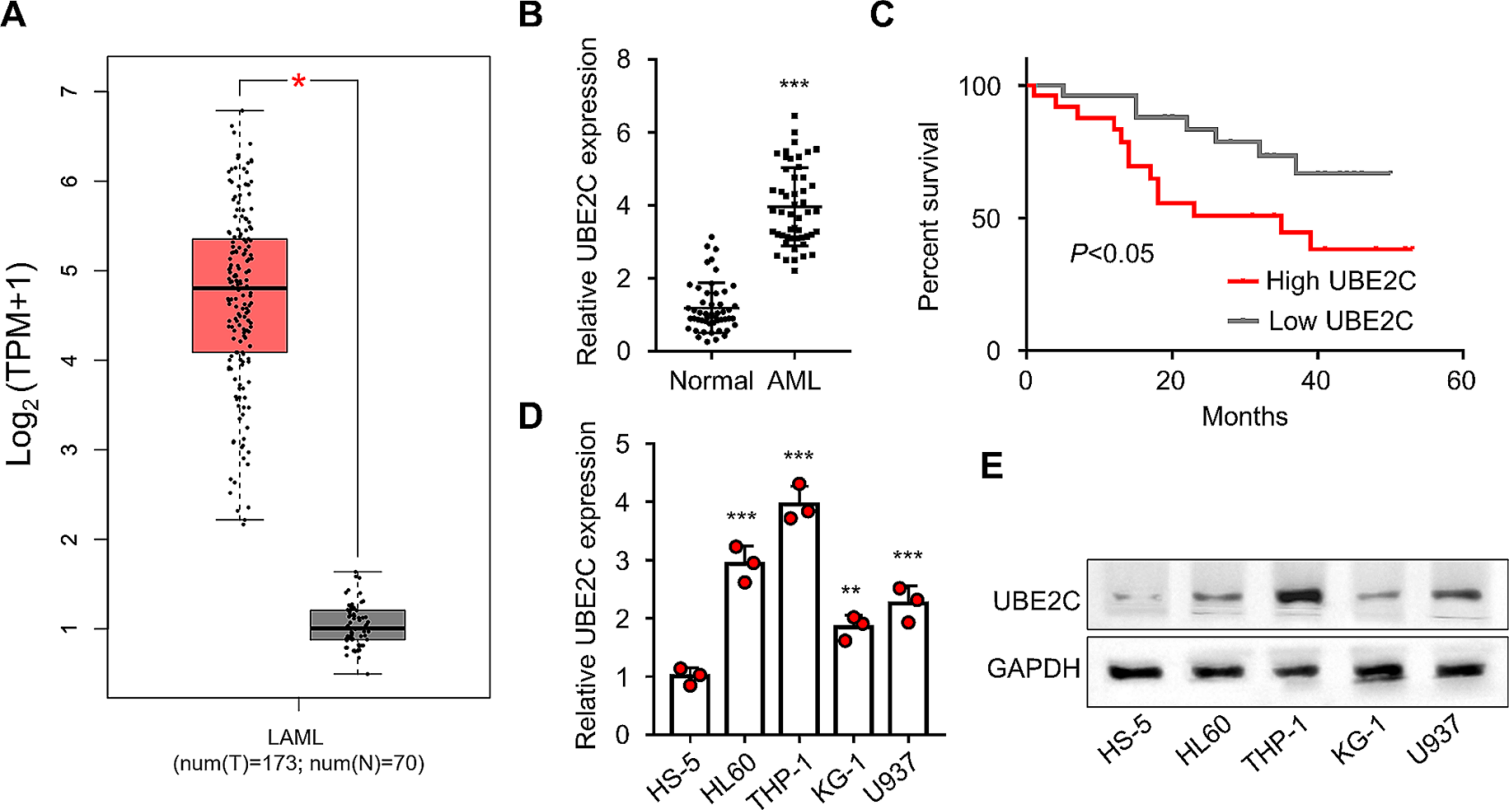
The leukemia cell lines show a high level of UBE2C expression. **(A)** GEPIA analysis indicates the UBE2C expression in the TCGA database. **(B)** The qRT-PCR analysis presents the UBE2C expression levels in the peripheral blood of 50 AML and 50 normal subjects. **(C)** KM-plotter displays the plotted survival curves of AML patients with high- and low-UBE2C expression groups. **(D)** The qRT-PCR analysis presents the UBE2C expression levels in AML cells and the human bone marrow stromal cell line, HS-5. **(E)** The WB analysis displays the expression levels of UBE2C protein in AML cell lines and the human bone marrow stromal cell line, HS-5. ** indicates *P* < 0.01 compared with Normal and HS-5 groups; *** represents *P* < 0.001 compared with HS-5 group

To analyze the expression levels of UBE2C, we utilized the GEPIA database from TCGA (http://gepia.cancer-pku.cn/). The data from GEPIA analysis indicated that UBE2C was highly expressed in acute leukemia samples (*P* < 0.01, Fig. 1A). We further collected 50 pairs of AML samples and matched normal samples to validate UBE2C expression pattern. Specifically, the AML samples exhibited a significantly higher expression of UBE2C compared to the normal samples (*P* < 0.001, Fig. 1B). Moreover, patients with high UBE2C expression had a poorer prognosis in comparison to those with low UBE2C expression (*P* < 0.05, Fig. 1C). Additionally, the qRT-PCR and WB analyses demonstrated a significant increase in mRNA and protein expressions of UBE2C in AML cell lines (HL60, THP-1, U937, and KG-1 cells) when compared to HS-5 control (*P* < 0.05, Fig. 1D–E). These results demonstrated the overexpression of UBE2C in AML patients.

### UBE2C knockdown inhibits proliferation and induced apoptosis in leukemic cells

**Fig. 2:**
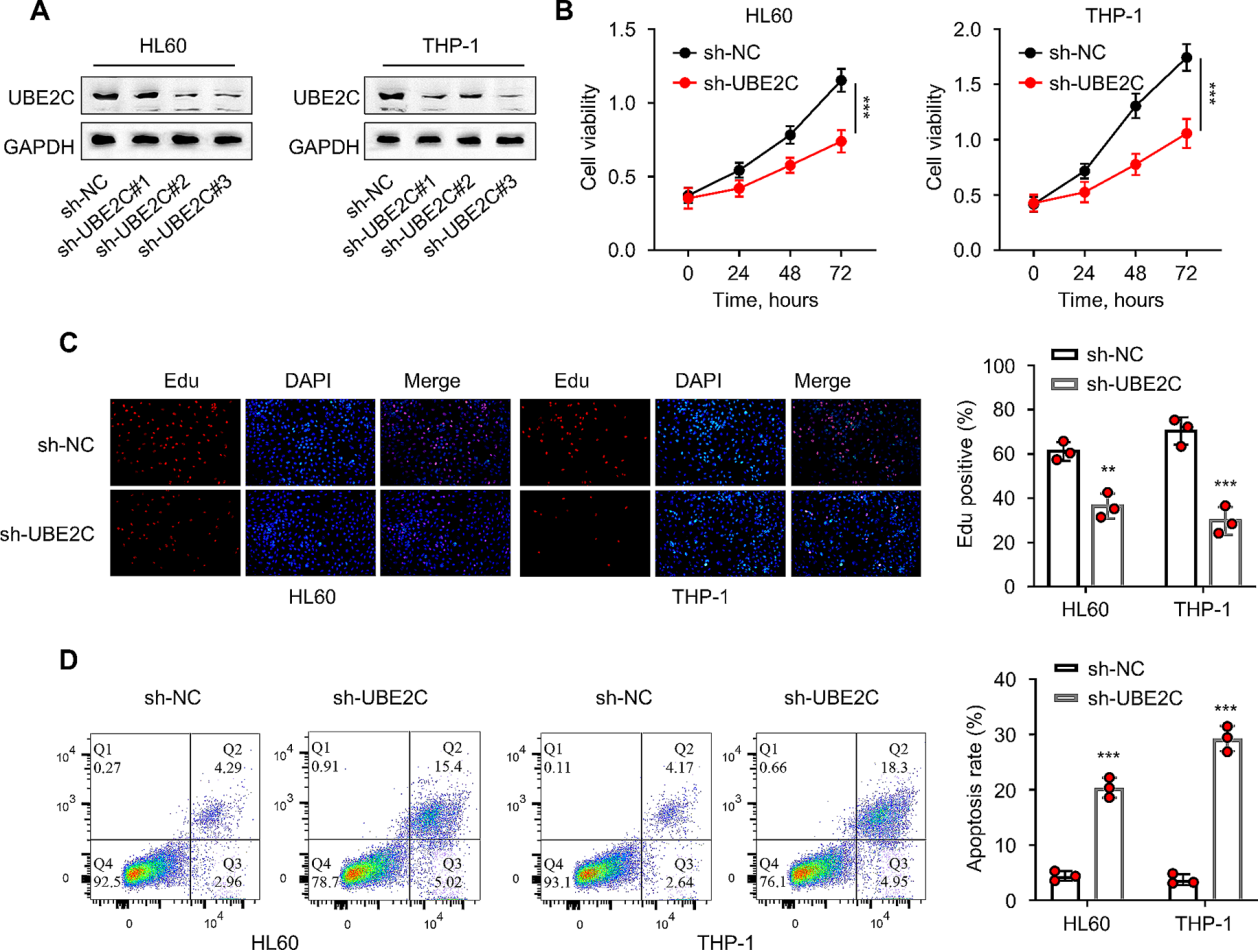
UBE2C knockdown inhibits the proliferation and induced apoptosis in leukemic cells. **(A)** The WB analysis detects the interference efficiency of UBE2C. **(B)** The CCK-8 assay detects the viability of HL60 and THP-1 cells at 0, 24, 48, and 72 h. **(C)** EdU staining assay detects the proliferation levels of HL60 and THP-1 cells. **(D)** The flow cytometry technique determines the apoptosis rate of HL60 and THP-1 cells. ** represents *P* < 0.01 compared with the sh-NC group; *** indicates *P* < 0.001 compared with the sh-NC group

Since UBE2C is significantly up-regulated in AML cells, we next applied sh-RNAs to reduce its expression to perform loss-of-function analysis. UBE2C protein levels were reduced in HL60 and THP-1 cells after the knockdown with sh-UBE2C#1, sh-UBE2C#2, and sh-UBE2C#3 when compared to sh-NC group (*P* < 0.05, Fig. 2A). Among the three sh-RNAs, sh-UBE2C#3 showed the strongest silencing effect and it was selected for the subsequent experiments. The CCK-8 cell viability assay showed that the relative viability of HL60 and THP-1 cells in the sh-UBE2C group was significantly reduced compared with the sh-NC group (*P* < 0.001, Fig. 2B). In addition, the qualitative proliferation analysis by EdU staining also revealed a restricted cell proliferation upon UBE2C knockdown (*P* < 0.01, Fig. 2C). In contrast, there was an increase in apoptotic events in the sh-UBE2C group (*P* < 0.001, Fig. 2D). These findings indicate that the knockdown of UBE2C could significantly inhibit the proliferation and induce apoptosis in AML cells.

### UBE2C knockdown promotes ferroptosis induction in leukemic cells

**Fig. 3:**
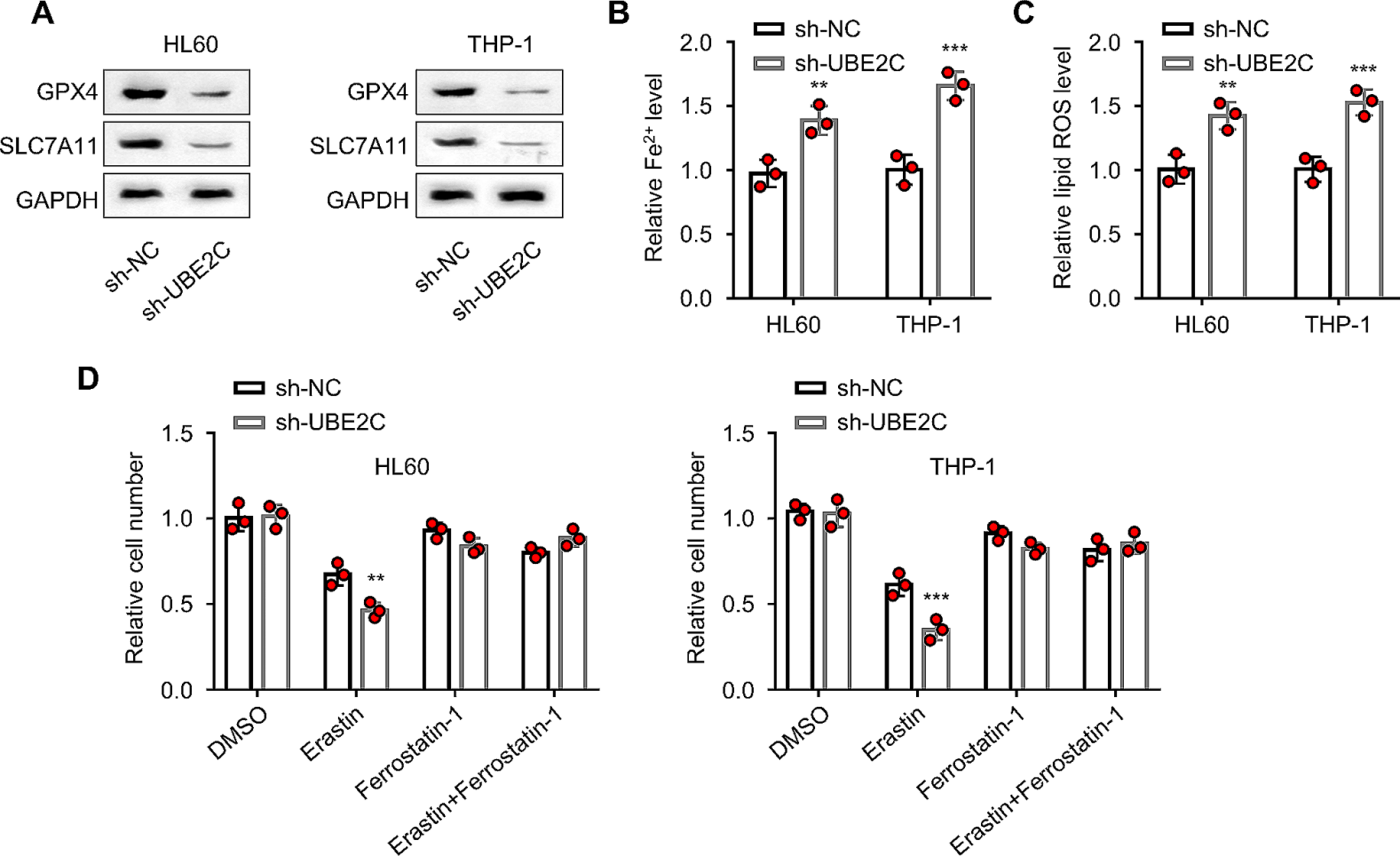
UBE2C knockdown induces ferroptosis in leukemic cells. **(A)** The WB analysis detects the expression levels of GPX4 and SLC7A11 proteins in HL60 and THP-1 cells. **(B)** The iron assay kit determines the Fe^2+^ levels in HL60 and THP-1 cells. **(C)** The flow cytometry analysis determines ROS levels in HL60 and THP-1 cells. **(D)** The CCK-8 method detects the relative cell viability in HL60 and THP-1 cells after different treatments (DMSO, erastin (1 µM), ferrostatin-1 (1 µM), and erastin + ferrostatin-1). Cells were treated for 48 h before analysis and data in each group was normalized against the DMSO control. ** signifies *P* < 0.01 compared with sh-NC group; *** indicates *P* < 0.001 compared with sh-NC group

We next wondered whether silencing UBE2C impinges on ferroptosis sensitivity in AML cells. Compared with the sh-NC group, the protein levels of GPX4 and SLC7A11 (anti-ferroptosis proteins) were significantly downregulated in the sh-UBE2C group of HL60 and THP-1 cells (*P* < 0.05, Fig. 3A). Further, the relative cellular levels of Fe^2+^ and ROS were significantly increased after UBE2C silencing (*P* < 0.01, Fig. 3B–C). We further applied erastin (a ferroptosis chemical inducer) and ferrostatin-1 (a ferroptosis inhibitor) in HL60 and THP-1 cells, with or without UBE2C knockdown. Erastin treatment decreased the cell viability in both sh-NC and sh-UBE2C groups, while sh-UBE2C displayed a significantly lower level of cell viability (*P* < 0.01, Fig. 3D). The application of ferrostatin-1 rescued cell viability in both sh-NC and sh-UBE2C groups after erastin treatment. These results suggest that UBE2C knockdown could promotes ferroptosis sensitivity in AML cells.

### UBE2C knockdown sensitizes ferroptosis in a proteasome-dependent manner

**Fig. 4:**
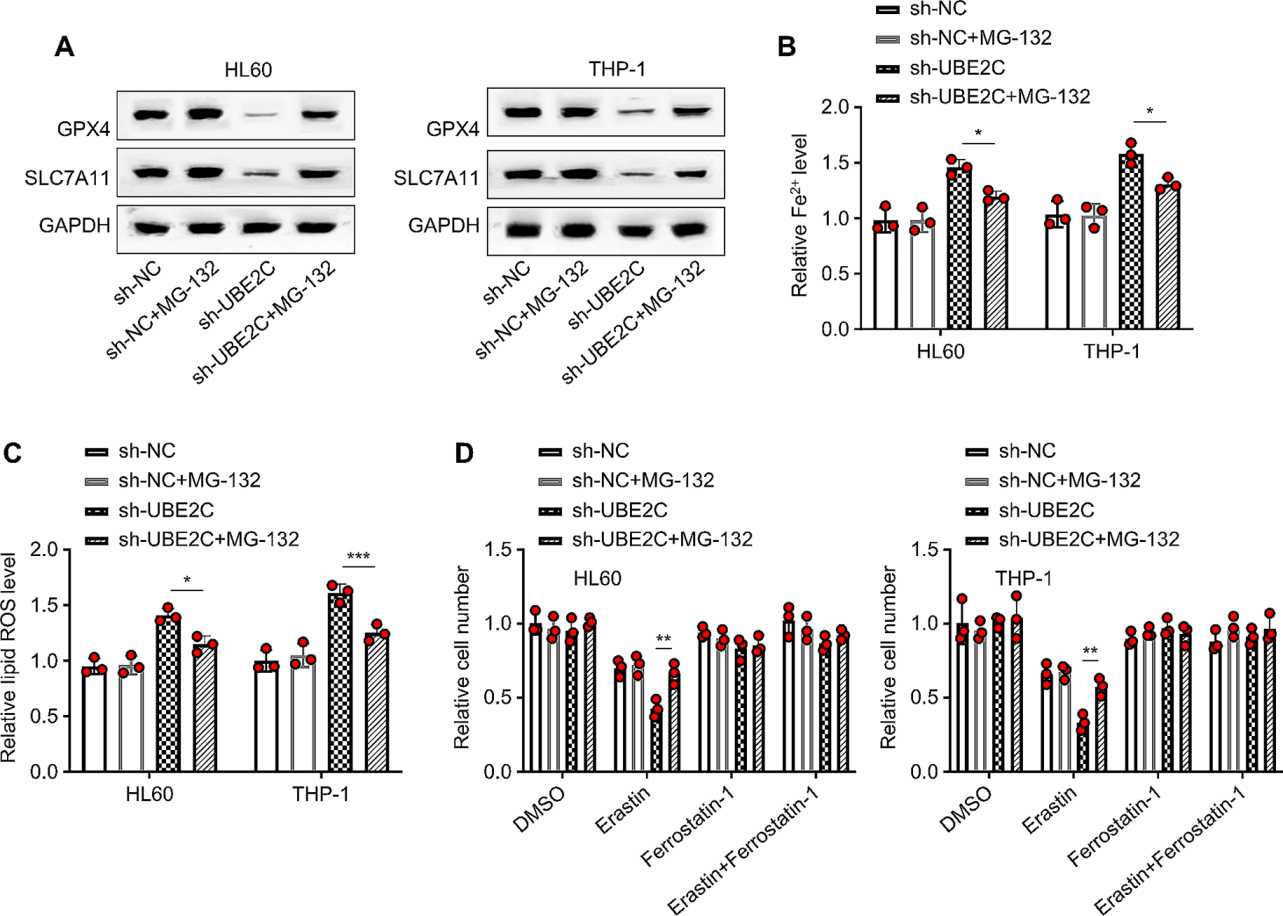
UBE2C knockdown sensitizes ferroptosis in a proteasome-dependent manner. **(A)** The WB analysis detects the expression levels of GPX4 and SLC7A11 proteins in HL60 and THP-1 cells. **(B)** The iron assay kit determines the Fe^2+^ levels in HL60 and THP-1 cells. **(C)** The flow cytometry analysis determines ROS levels in HL60 and THP-1 cells. **(D)** The CCK-8 method detects the relative cell viability in HL60 and THP-1 cells after different treatments (DMSO, erastin (1 µM), ferrostatin-1 (1 µM), and erastin + ferrostatin-1). MG-132 was applied at 1 µM. Cells were treated for 48 h before analysis and data in each group was normalized against the DMSO control. ** signifies *P* < 0.01 comparison between sh-UBE2C and sh-UBE2C + MG-132.

Since we observed that UBE2C knockdown led to the decreased protein levels of SLC7A11 and GPX4, it is possible that these anti-ferroptosis proteins are degraded by proteasome. To test this hypothesis, we introduced MG-132 (a well-established proteasome inhibitor) to the cells after UBE2C knockdown. We observed that proteasome inhibition restored SLC7A11 and GPX4 levels upon UBE2C knockdown in both HL60 and THP-1 cells (*P* < 0.01, Fig. 4A). Besides, the application of proteasome inhibitor reduced cellular ROS and Fe^2+^ levels after UBE2C knockdown (*P* < 0.01, Fig. 4B–C), and also suppressed Erastin-induced cell death upon UBE2C knockdown (*P* < 0.01, Fig. 4D). Thus, these results imply that UBE2C knockdown sensitizes ferroptosis of AML cells in a proteasome-dependent manner.

### UBE2C affects the proliferation of leukemia cells by regulating PI3K/AKT pathway

**Fig. 5:**
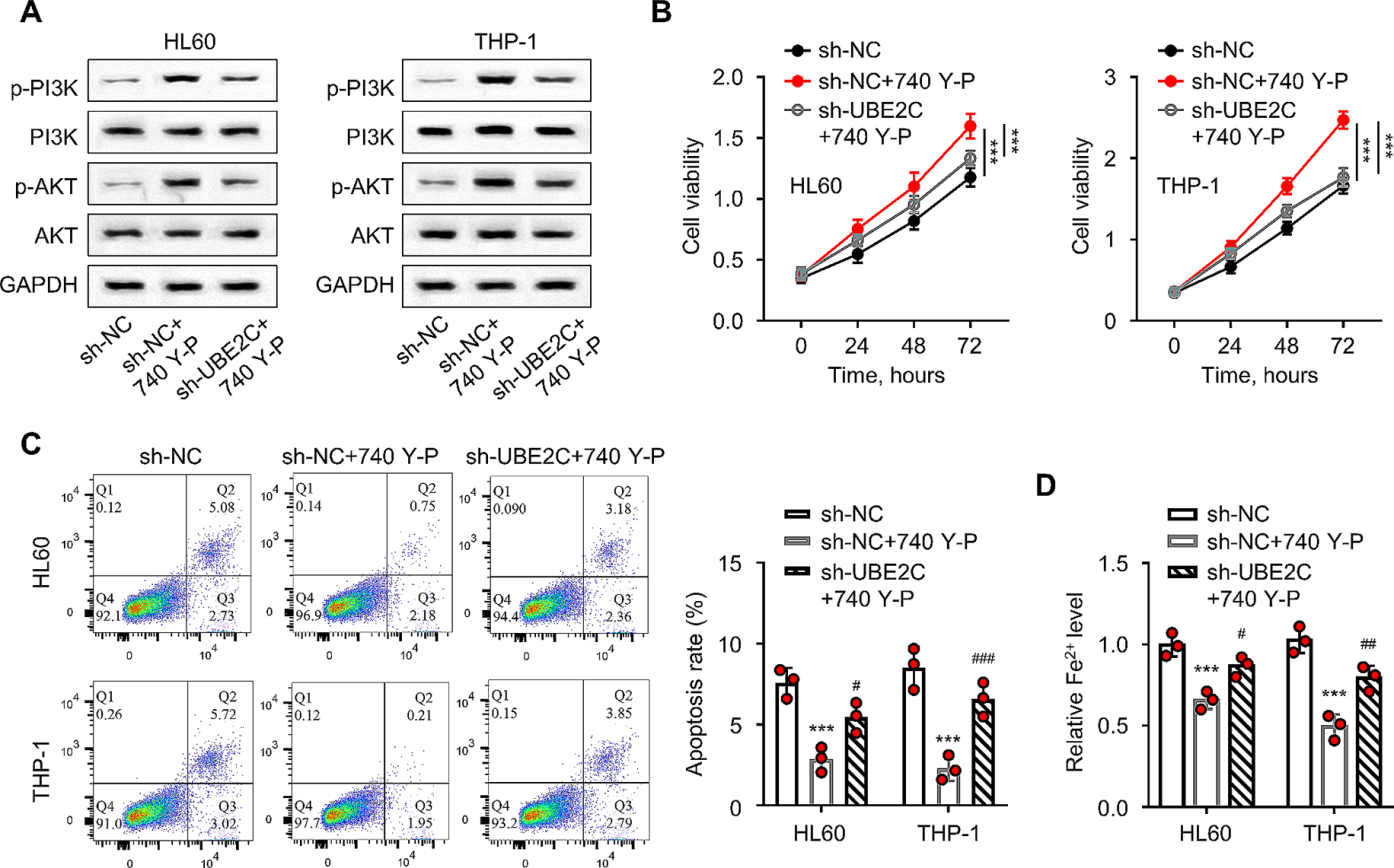
UBE2C affects proliferation and ferroptosis in leukemic cells by regulating the PI3K/AKT pathway. **(A)** The WB analysis detects the expression levels of p-PI3K, PI3K, and p-AKT, AKT proteins in HL60 and THP-1 cells. **(B)** The CCK-8 method detects the viability of HL60 and THP-1 cells after incubation for 0, 24, 48, and 72 h. **(C)** Flow cytometry detects the apoptosis levels of HL60 and THP-1 cells after different treatments. **(D)** The iron assay kit determines Fe^2+^ levels in HL60 and THP-1 cells. 740 Y-P was applied at 5 µM. ** signifies *P* < 0.01 compared with the sh-NC treatment group; *** indicates *P* < 0.001 compared with the sh-NC treatment group; # represents *P* < 0.05 compared with the sh-NC + 740 Y-P treatment group; ## presents *P* < 0.01 compared with the sh-NC + 740 Y-P treatment group

Furthermore, the mechanism of action of UBE2C in the HL60 and THP-1 cells was explored. We applied a PI3K/AKT pathway activator (740 Y-P) to examine whether the activation of PI3k/AKT signaling coudl rescue the effect of UBE2C knockdown. The protein levels of p-PI3K and p-AKT proteins were significantly increased in HL60 and THP-1 cells in the sh-NC + 740 Y-P group compared to the sh-NC group. However, the expression levels of p-PI3K and p-AKT were significantly downregulated in the sh-UBE2C + 740 Y-P treatment group, when compared to the sh-NC + 740 Y-P treatment group (*P* < 0.05, Fig. 5A). Moreover, the cell viability of HL60 and THP-1 cells in the sh-NC + 740 Y-P treatment group were significantly increased, but the sh-UBE2C + 740 Y-P treatment group showed a reduced viability (*P* < 0.001, Fig. 5B). In addition, the apoptosis rates and Fe^2+^ levels were significantly decreased in the sh-NC + 740 Y-P treatment group compared with the sh-NC group. In contrast, the sh-UBE2C + 740 Y-P treatment group displayed a higher level of apoptosis and an increase in Fe^2+^ level compared with the sh-NC + 740 Y-P treatment group (*P* < 0.05, Fig. 5C–D). These findings suggest that UBE2C might affect the proliferation and ferroptosis of AML cells by regulating the PI3K/AKT pathway.

### UBE2C knockdown suppresses tumor formation of leukemia cells in vivo

**Fig. 6:**
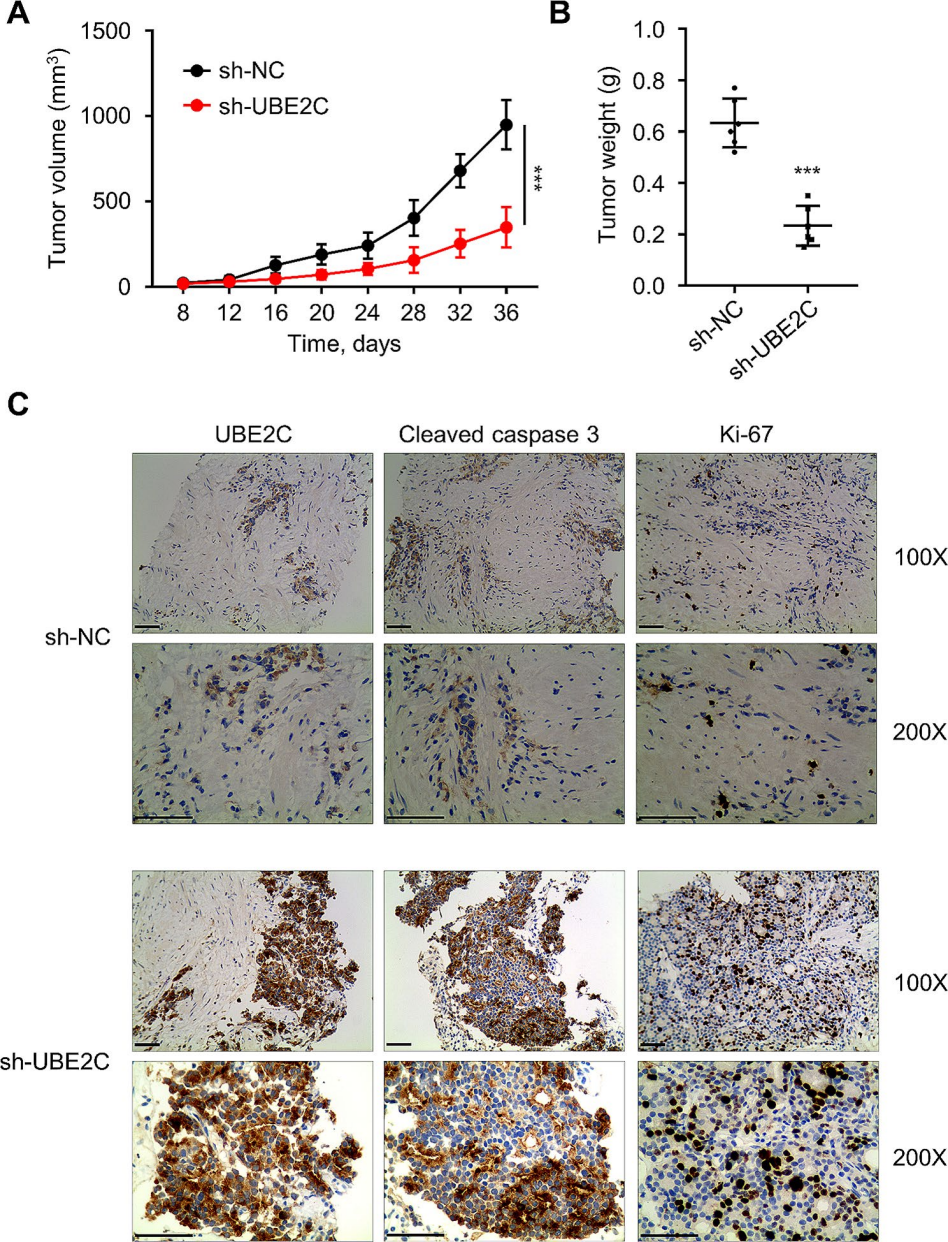
UBE2C knockdown inhibits the proliferation of leukemia cells in vivo. **(A)** The graph shows the subcutaneous tumor volume in nude mice after 4, 8, 12, 16, 20, 24, 28, 32, and 36 days. **(B)** The graph presents the tumor mass in nude mice treated with sh-UBE2C. **(C)** The images show the immunohistochemical detection of expression levels of UBE2C, KI-67, and cleaved-caspase 3 in tumor tissues. *** signifies *P* < 0.001 compared with the sh-NC group

Considering the in vitro results, the effect of UBE2C silencing in the tumor formation of AML cells was explored in tyhe nude mice. The volume and mass of tumors in the nude mice of the sh-UBE2C group were significantly reduced compared with the sh-NC group (*P* < 0.001, Fig. 6A–B). Moreover, the IHC staining in the tumor tissues revealed a decreased expression of Ki-67 (cell proliferation marker) and an increased expression of cleaved-caspase 3 (apoptosis marker) in sh-UBE2C group (Fig. 6C). These data altogether indicate that the UBE2C knockdown could inhibit the growth of subcutaneous transplanted AML cells in nude mice, suggesting the oncogenic function of UBE2C.

## Discussion

AML has become the most common type of acute leukemia in adults. It is characterized by the accumulation of mature primitive cells due to insufficient maturation, uncontrolled proliferation, dysregulated apoptosis, and restriction of normal hematopoietic functionalities [21]. Currently, AML treatment primarily relies on chemotherapy, which unfortunately affects normal cells and leads to adverse effects. As a result, there is a need to develop new therapeutic strategies to address these issues. Several advancements have been made in exploring different treatment options and their underlying mechanisms. Several reports indicated that UBE2C was highly expressed in various cancers [16, 17, 22]. The current study demonstrated that UBE2C expression was significantly upregulated in AML, and a high level of UBE2C expression was linked with a poor prognosis in AML patients. The knockdown of UBE2C significantly suppressed the proliferation of AML cells and promoted apoptosis. In addition, it was observed that UBE2C knockdown promoted Erastin-induced ferroptosis. Notably, UBE2C silencing also inhibited tumor growth of AML cells in nude mice. Together, our finding indicate that UBE2C functions as an oncogene to support the hyper-proliferation and protects against cell death in AML cells.

UBE2C is an essential protein required to regulate mitotic cell cycle progression. Moreover, it has been demonstrated that UBE2C deregulation plays an important role in the development and malignant progression of various cancers [23]. Furthermore, Bose and colleagues [24] demonstrated that the downregulation of UBE2C could substantially increase the sensitivity of tumor cells to radiotherapy. In the current study, we revealed that UBE2C silencing not only promoted apoptosis in AML cells, but also sensitized AML cells to erastin-induced ferroptosis. These findings are in agreement with a previous report, in which UBE2C knockdown significantly impaired the malignant features of cervical squamous cell carcinoma cells [25]. Similarly, Kim and coworkers [26] showed that UBE2C overexpression could promote the proliferation of breast cancer cells, and UBE2C upregulation is linked with a poor prognosis. Nevertheless, whether UBE2C is overexpressed only in certain subtypes of leukemia warrants future investigation.

The initiation of ferroptosis relies on iron-mediated Fenton reaction, which generates free radicals to induce lipid peroxidation in the plasma membrane. The lipid peroxidation could be antagonized by glutathione peroxidase 4 (GPX4) [27]. Thus, the imbalance between Fe^2+^ accumulation and GPX4 activity can trigger ferroptosis in different pathophysiological conditions. Furthermore, accumulating evidence has implicated the protein ubiquitination system in regulating ferroptosis sensitivity through targeting various ferroptosis regulators [12, 13]. Accordingly, the present study showed that UBE2C knockdown significantly increased the intracellular accumulation of Fe^2+^ and ROS levels in AML cells, an effect depending on the activity of proteasome. In another study, the authors showed that arsenic trioxide induced cell death in drug-resistant leukemia stem cells through the upregulation of ROS [28]. Although elevated ROS plays a crucial role in tumor initiation and progression, the excessive accumulation of ROS incurs oxidative damages and triggers cell death in tumor cells [29]. Therefore, UBE2C knockdown in this study might inhibit AML cell proliferation through the generation of dreadful ROS intracellularly. Of note, the mechanism by which UBE2C knockdown triggers ROS production and sensitizes AML cells to ferroptosis needs future clarification.

The PI3K/AKT signaling pathway has been identified as a potential target for treating metastatic tumors [30]. A previous study reported that UBE2C could promote the progression of pancreatic cancer through the PI3K/AKT signaling pathway [31]. In another report, PD-L1 was found to promote the proliferation and survival of AML cells by activating the PI3K/AKT signaling pathway [32]. Liu and coworkers [33] demonstrated that targeting the PI3K/AKT/mTOR pathway significantly enhanced the anti-leukemic efficacy of venetoclax. These findings suggest that suppressing PI3K/AKT signaling pathway can be a potential strategy to impair the malignancy of AML cells [34, 35]. In our study, UBE2C knockdown could also significantly suppress the phosphorylation of PI3K and AKT proteins in AML cells. Our findings suggest that UBE2C knockdown undermines the malignancy of AML cells through targeting PI3K/AKT signaling pathway. Whether this effect accounts for the impact of UBE2C knockdown on ferroptosis susceptibility needs to be clarified in the future study.

## Conclusion

In summary, we reported an upregulation of UBE2C in AML samples and cell lines. UBE2C knockdown could inhibit the proliferation of AML cells, promoting apoptosis and sensitizing the cells to ferroptosis induction. Notably, our data suggest that UBE2C could potentially regulate the PI3K/AKT signaling pathway to affect the survival of AML cells. The mechanism by which UBE2C regulates ferroptosis sensitivity in leukemic cells warrants future investigations.

### Electronic supplementary material

Below is the link to the electronic supplementary material.

Supplementary Material 1

## Data Availability

The datasets generated during and/or analyzed during the current study are not publicly available, but are available from the corresponding author on reasonable request.
