# Supplementary material for: UBE2C promotes the proliferation of acute myeloid leukemia cells through PI3K/AKT activation
Source: BMC Cancer. 2024 Apr 18;24:497. doi: 10.1186/s12885-024-12212-x (PMC11027220; doi:10.1186/s12885-024-12212-x)
Supplement: Supplementary file 1 — Supplementary Material 1 [file 12885_2024_12212_MOESM1_ESM.docx]

Fig1E


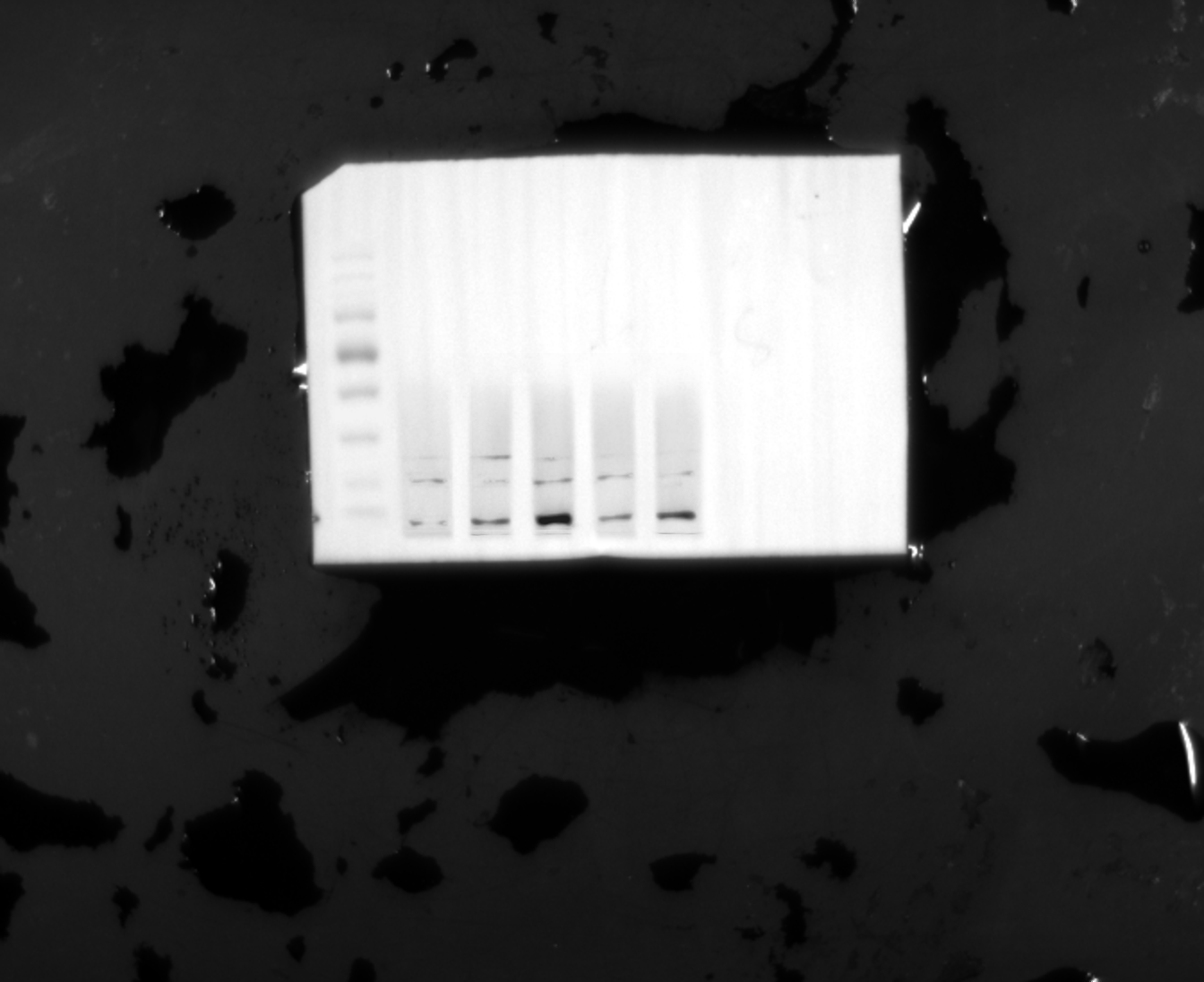


UBE2C


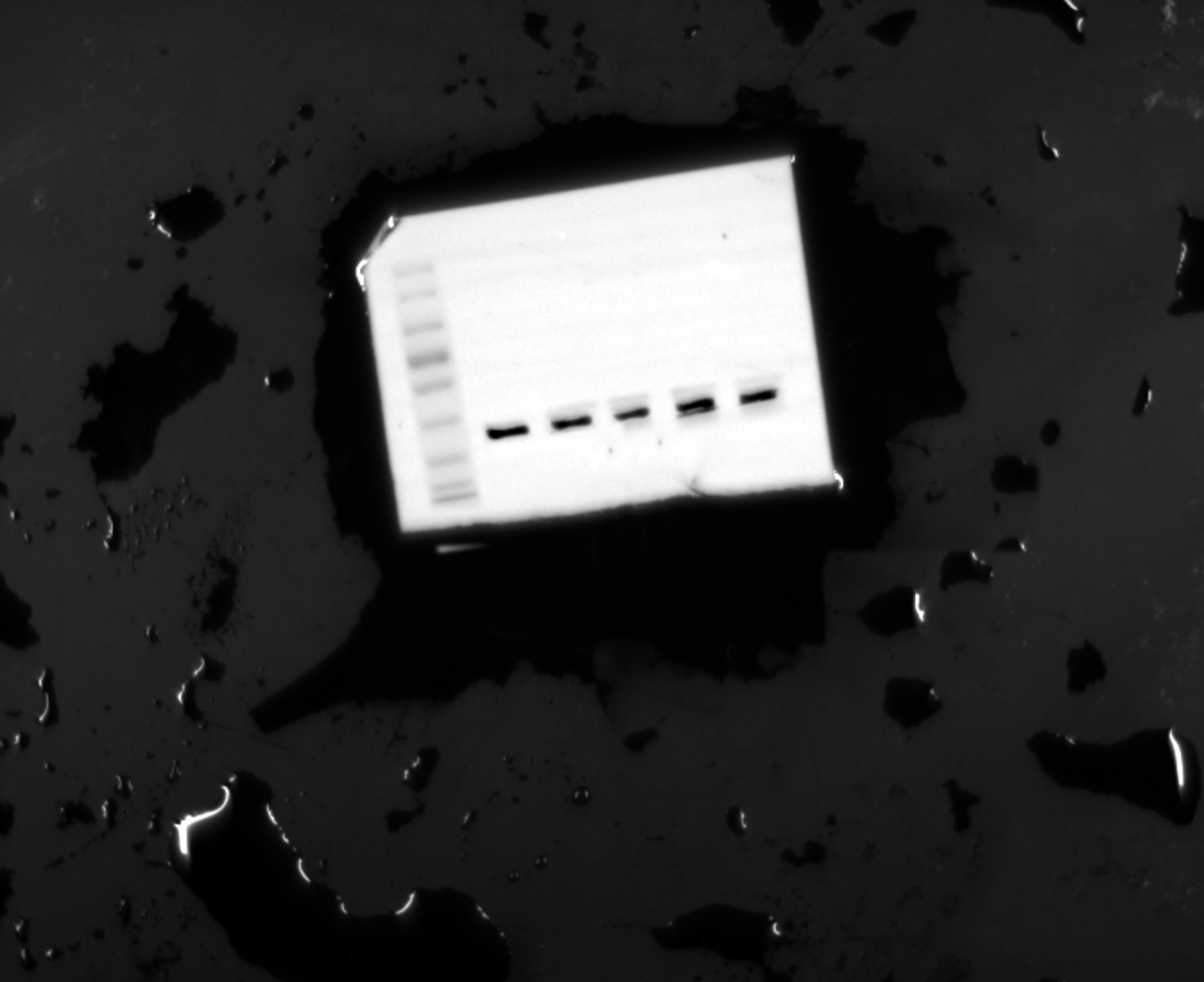


GAPDH

Fig2A


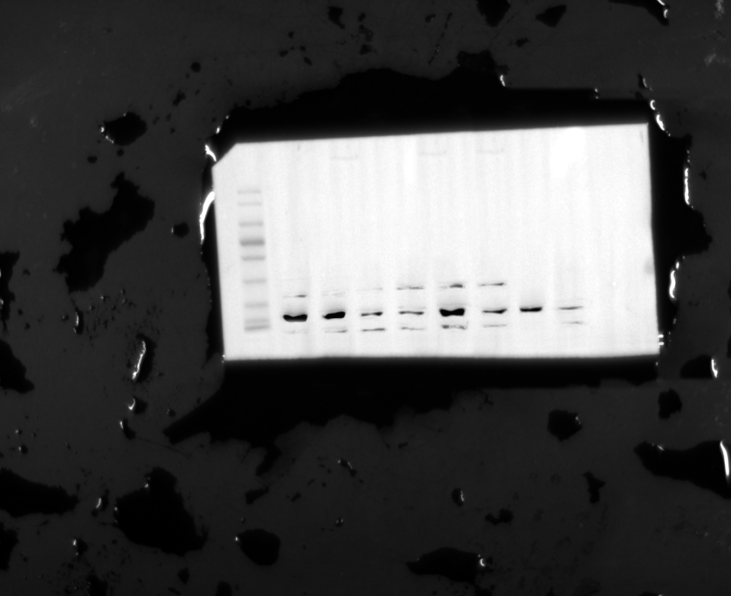


UBE2C


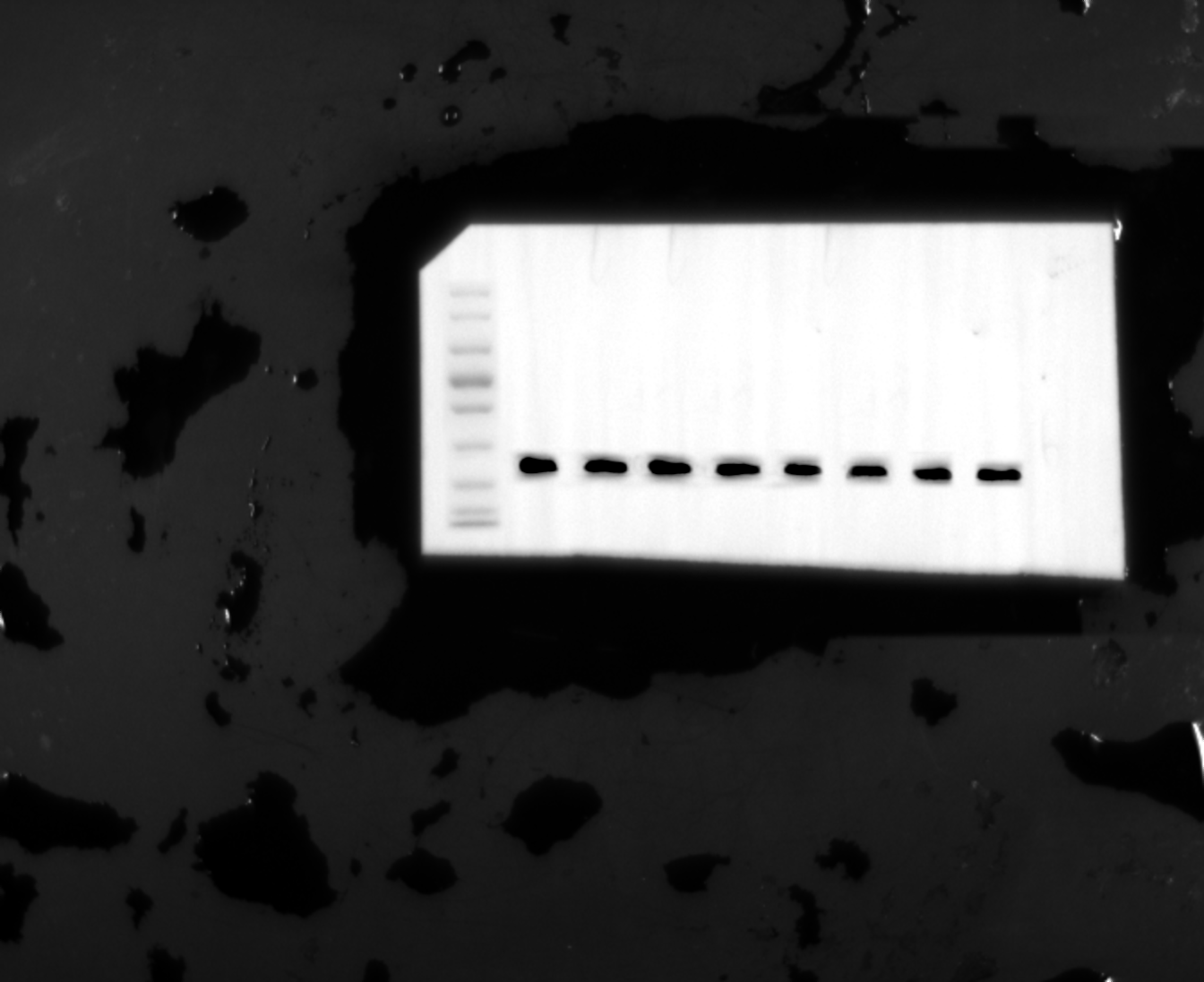


GAPDH

Fig3A


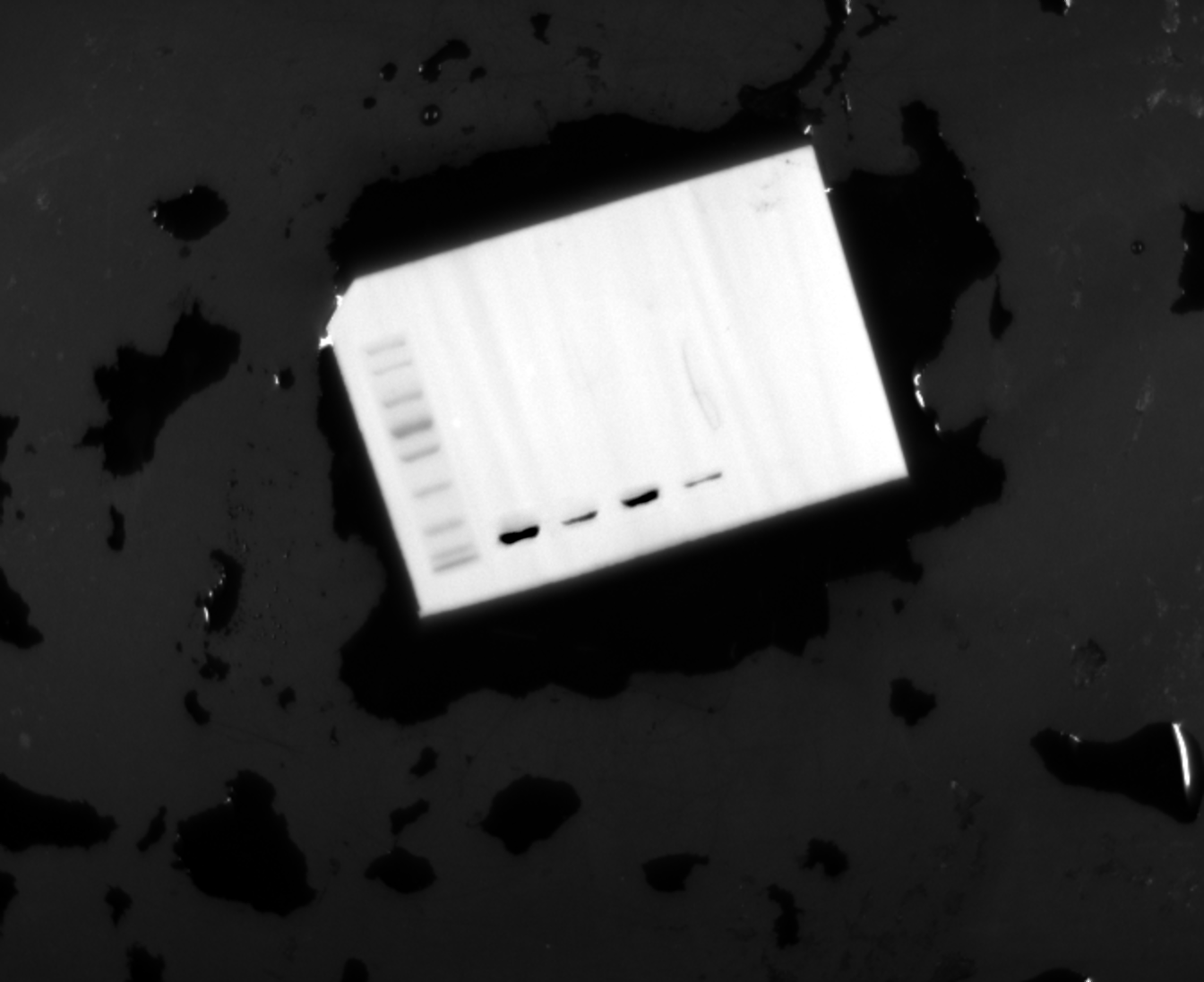


GPX4


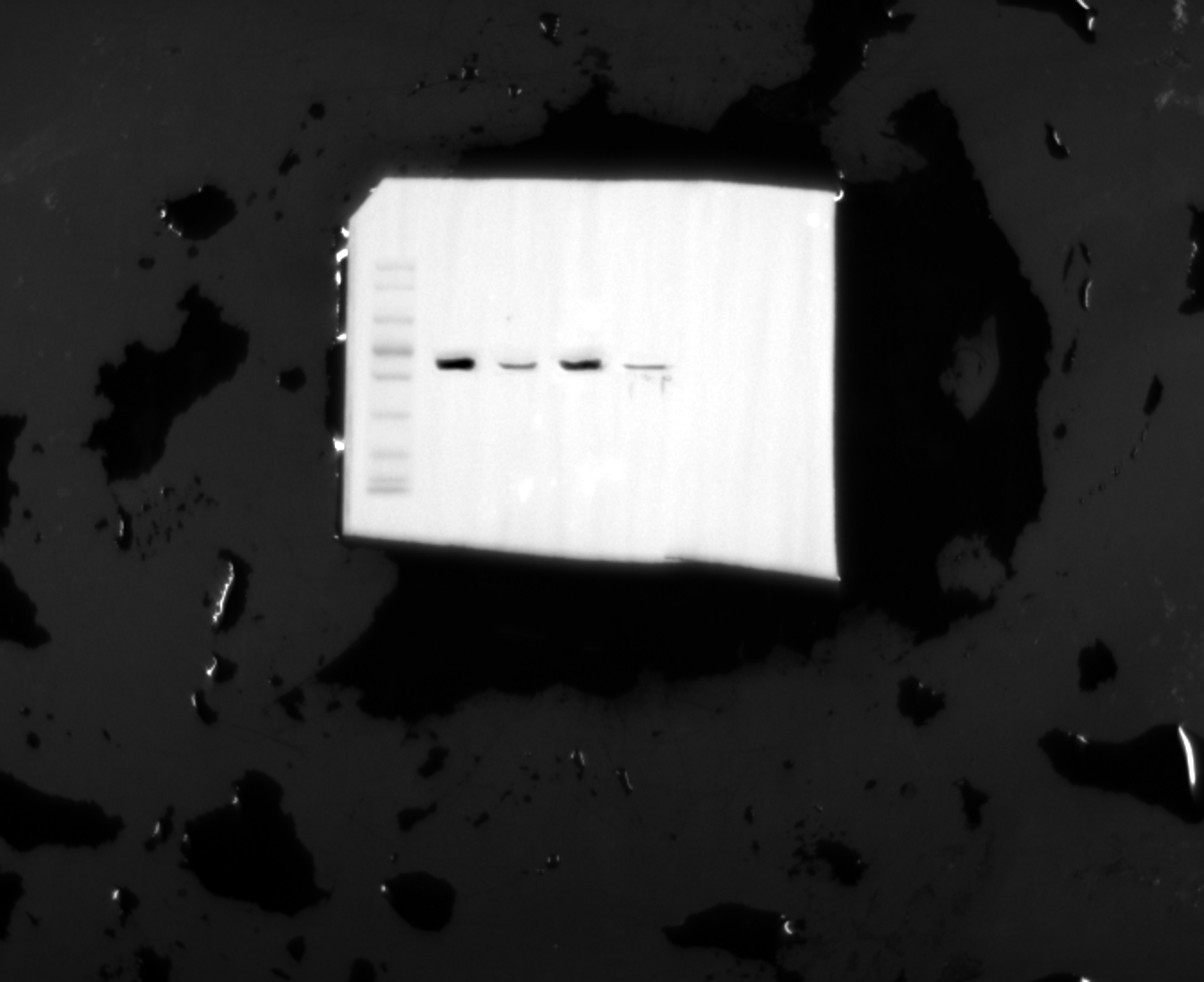


SLC7A11


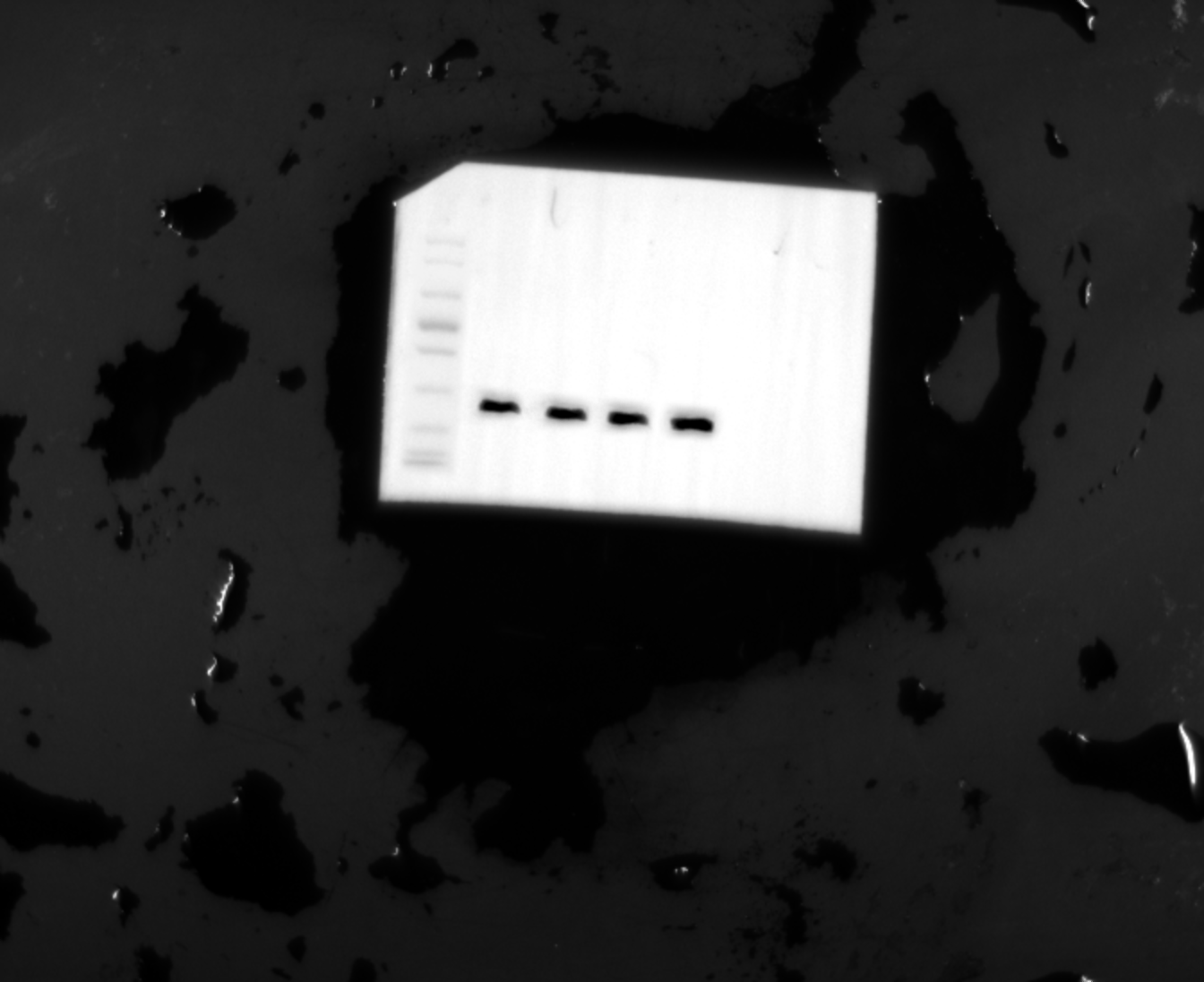


GAPDH

Fig4A


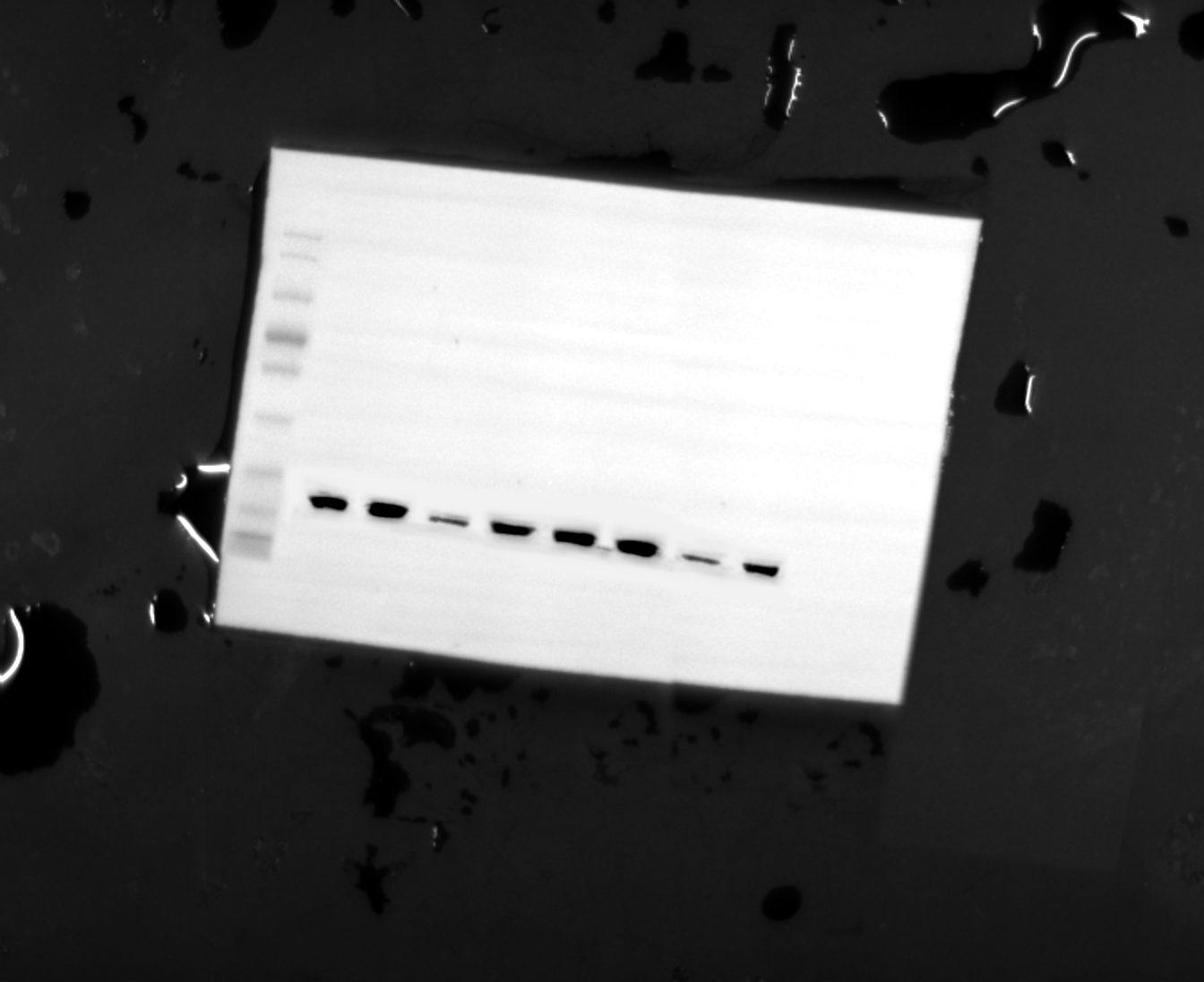

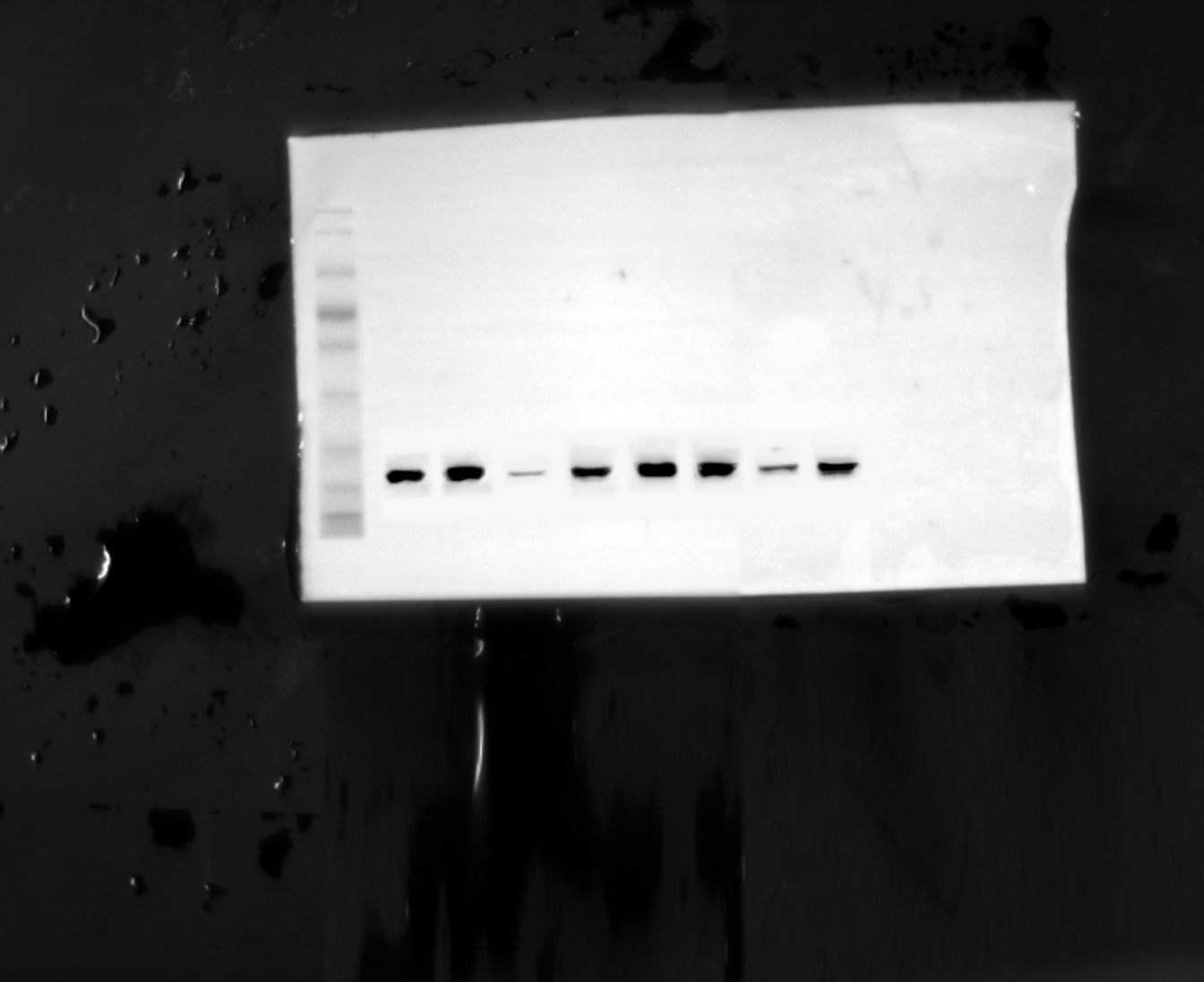


GPX4

SLC7A11


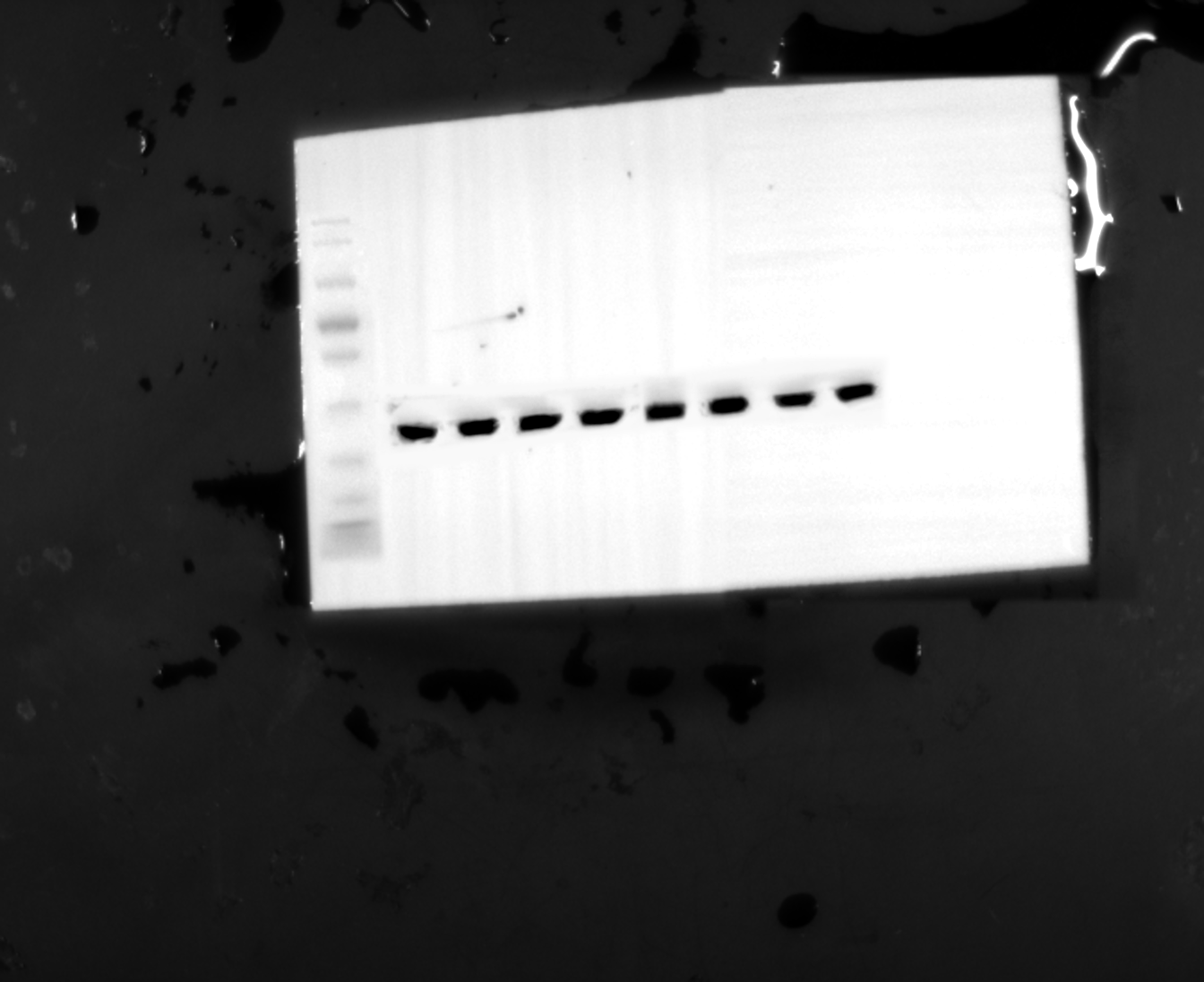


GAPDH

Fig5A


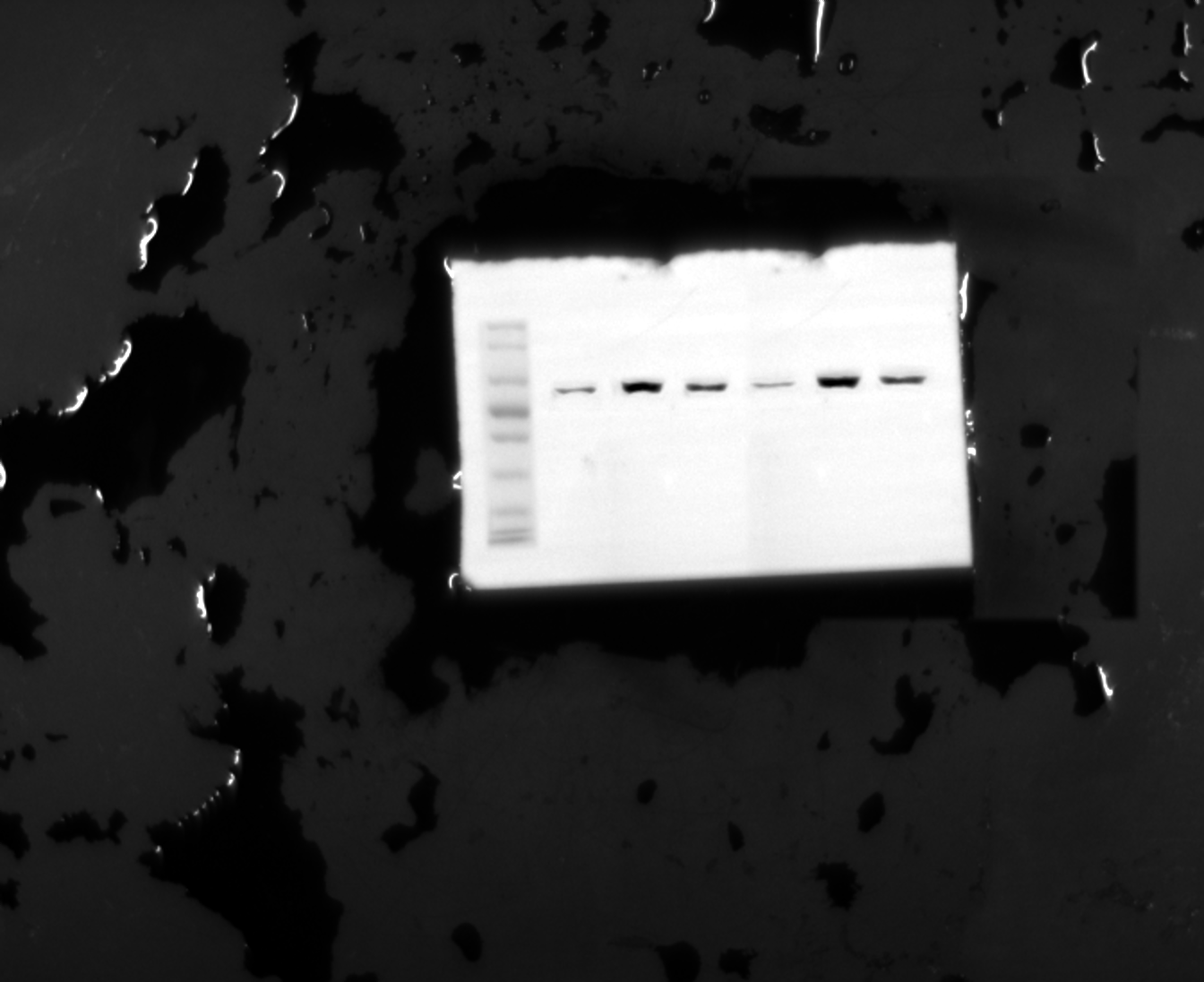


p-PI3K


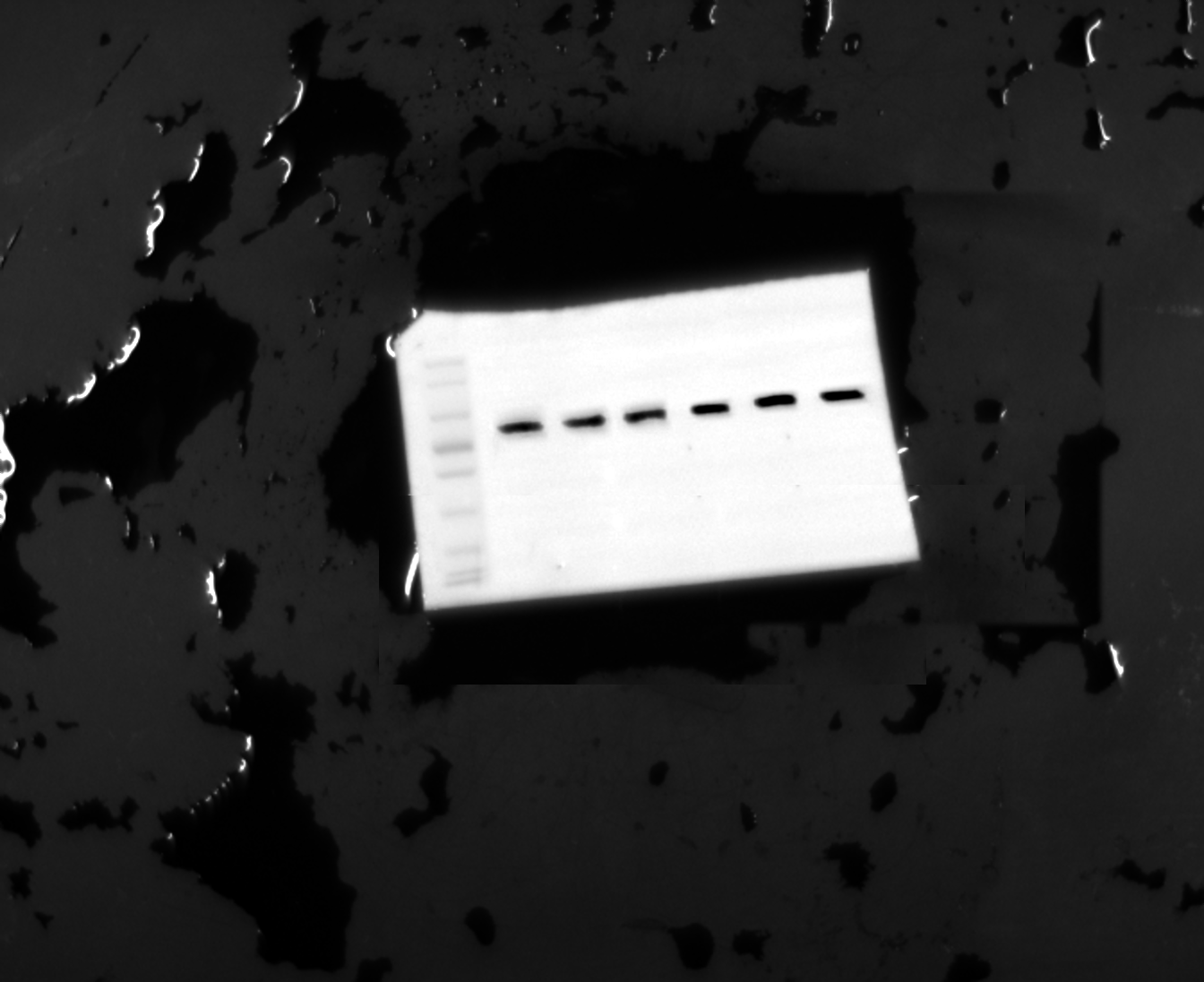


PI3K


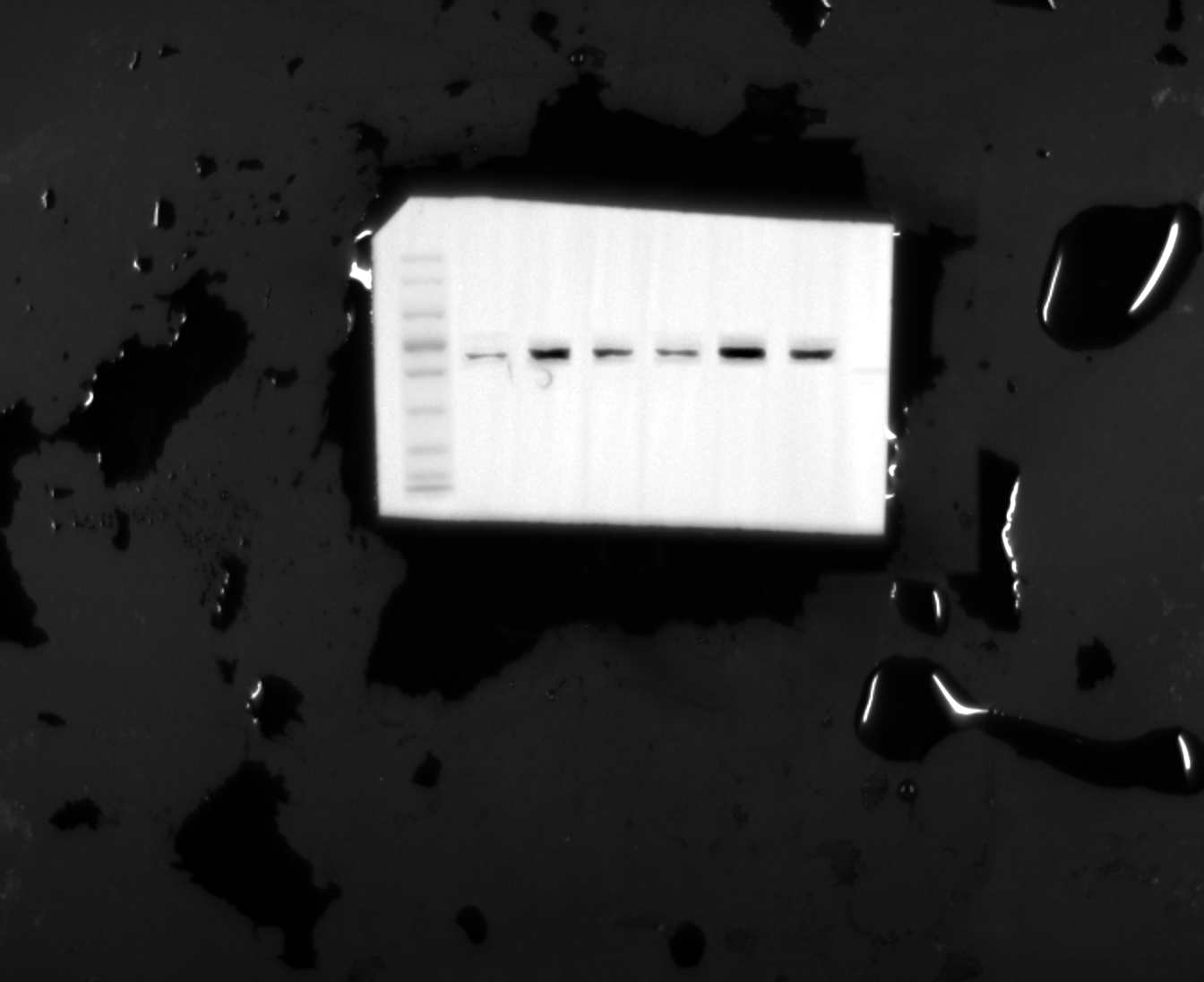


p-AKT


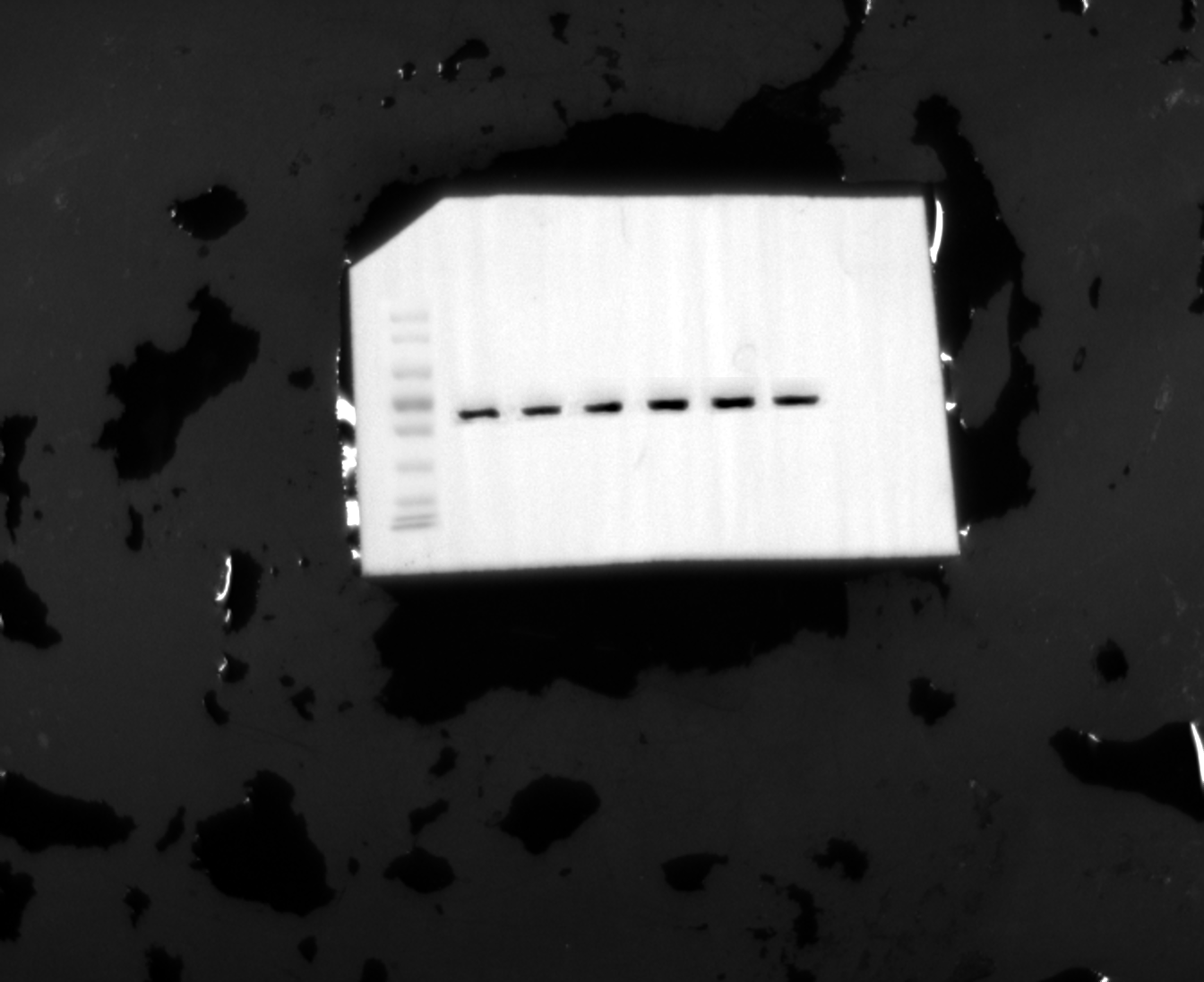


AKT


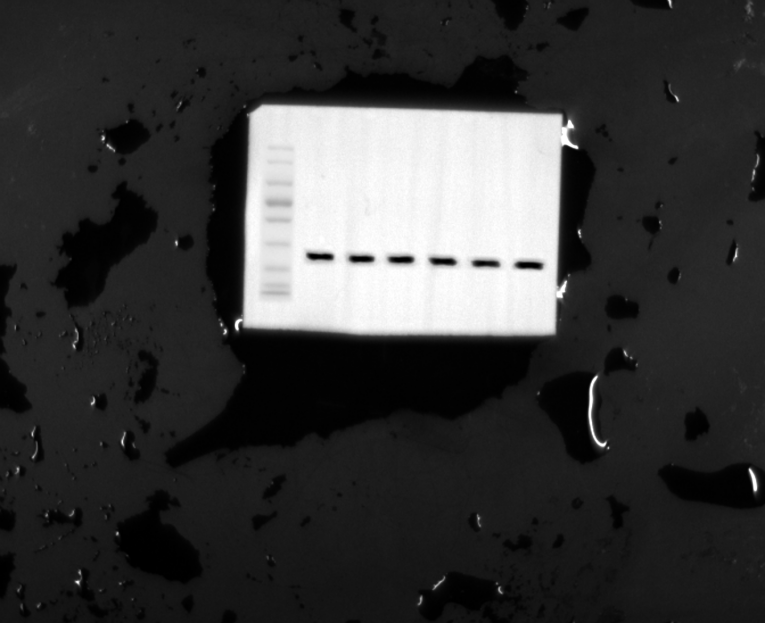


GAPDH
